# Supplementary material for: Development of E-ice-COLD-PCR assay combined with HRM analysis for Nucleophosmin1 gene mutation detection in acute myelogenous leukemia
Source: PLoS One. 2022 Sep 14;17(9):e0274034. doi: 10.1371/journal.pone.0274034 (PMC9473412; doi:10.1371/journal.pone.0274034)

**S1 Fig. The results of detecting *NPM1* gene mutations in total 83 patient samples using a standard PCR assay.**


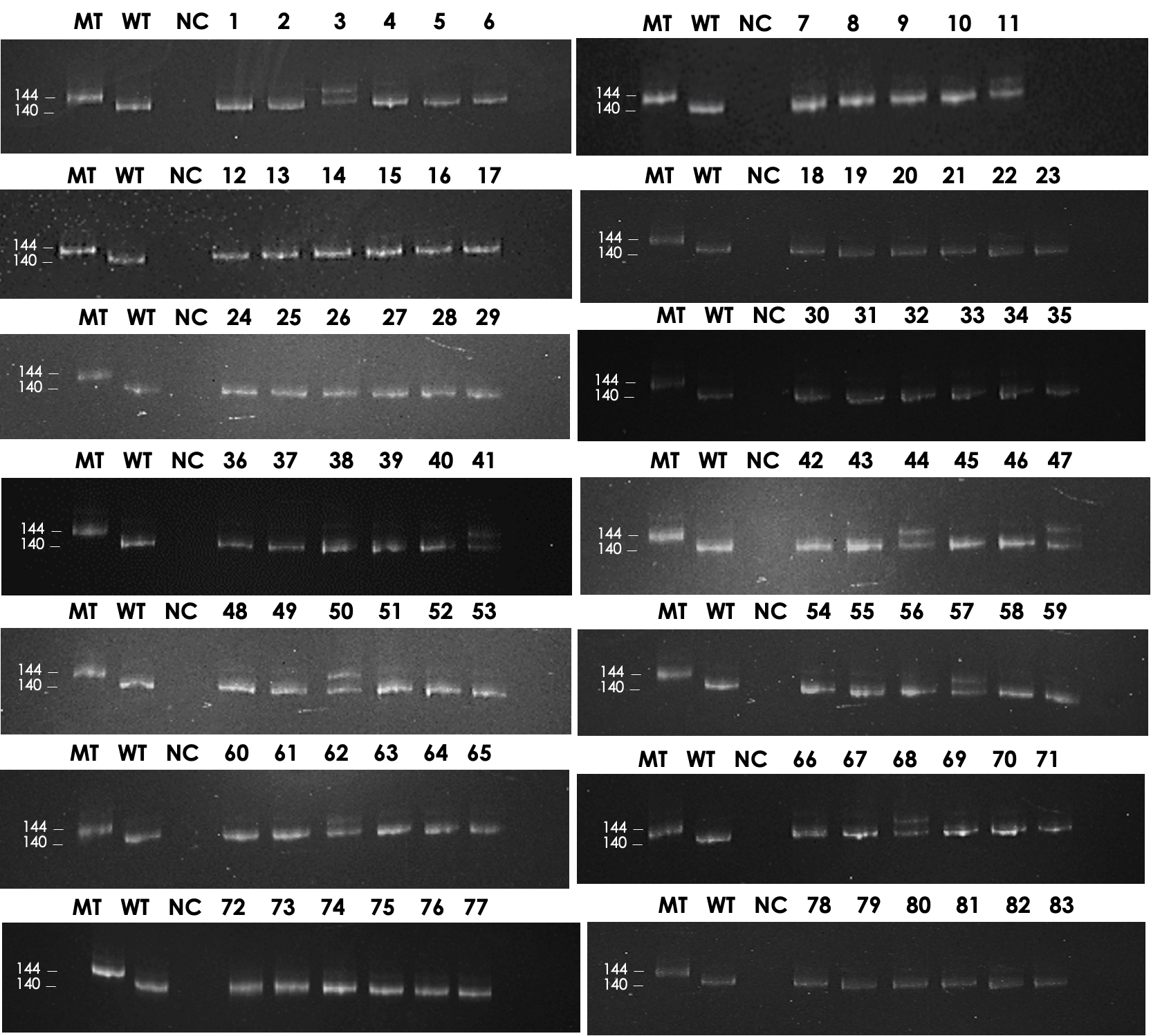

Supplement: S1 Fig — The PCR products are visualized on 10% polyacrylamide gel. MT indicates NPM1 mutated control and WT indicates NPM1 wild-type control. A double band indicates a heterogeneous mutation. (DOCX) [file pone.0274034.s001.docx]
