# Supplementary material for: Development of E-ice-COLD-PCR assay combined with HRM analysis for Nucleophosmin1 gene mutation detection in acute myelogenous leukemia
Source: PLoS One. 2022 Sep 14;17(9):e0274034. doi: 10.1371/journal.pone.0274034 (PMC9473412; doi:10.1371/journal.pone.0274034)
Supplement: S1 Data — (DOCX) [file pone.0274034.s003.docx]

**Supplementary data**

1. **The results of detecting *NPM1* gene mutations in total 83 patient samples using a standard PCR assay.** The PCR products are visualized on 10% polyacrylamide gel. MT indicates *NPM1* mutated control and WT indicates *NPM1* wild-type control. A double band indicates a heterogeneous mutation.


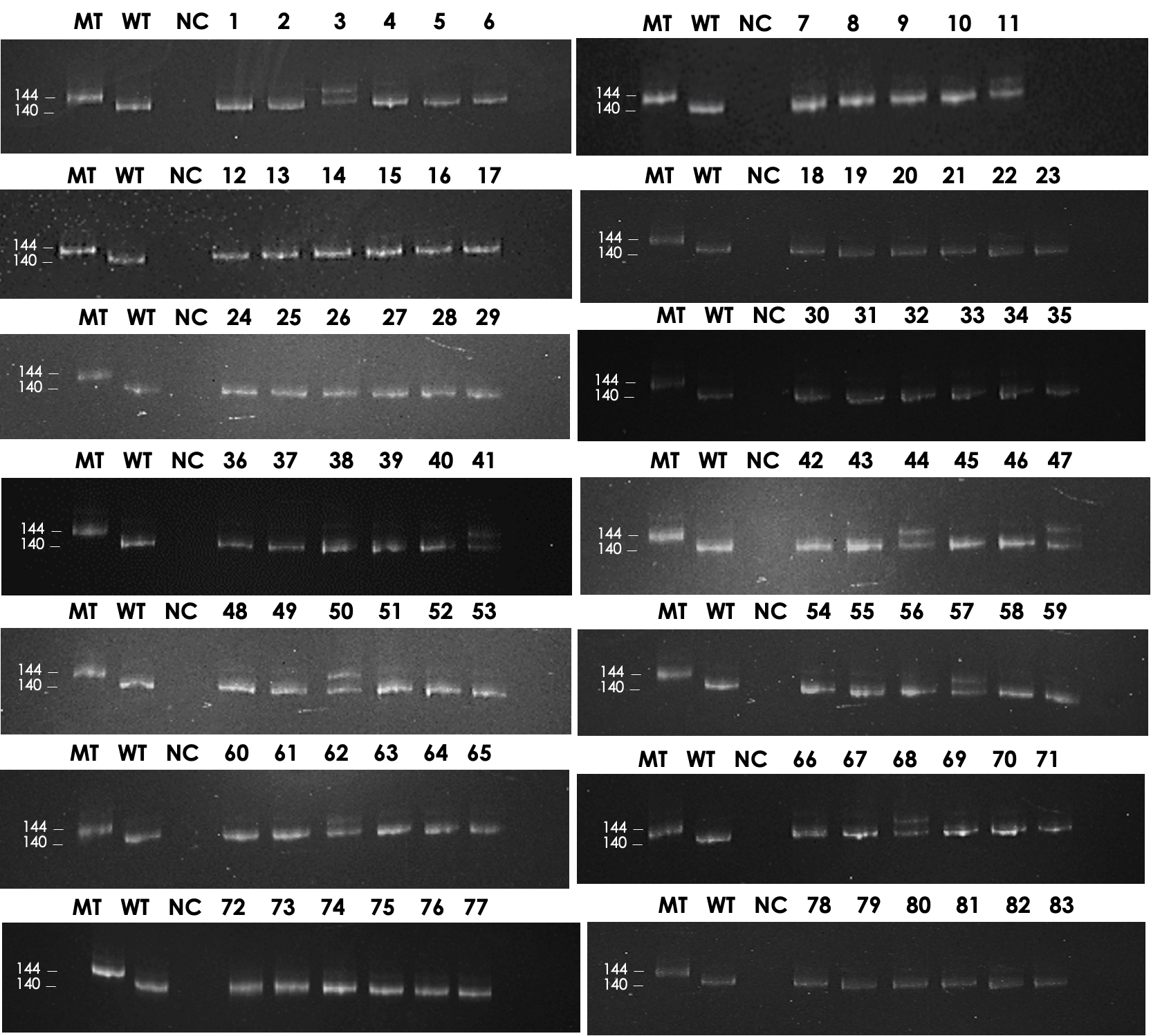


1. **Detecting *NPM1* gene mutations by HRM analysis.** Detecting *NPM1* gene mutations using the E-ice-COLD-PCR assay and generating the data into HRM difference curves. Red line indicates mutant control. Green line indicates wild-type control. The wild-type samples present the peak at the same location as the wild-type control, and the mutant samples present the peak at the same location as the mutant control.


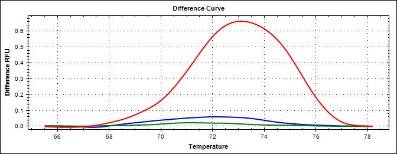


HRM result of sample no.1 represented wild-type *NPM1*


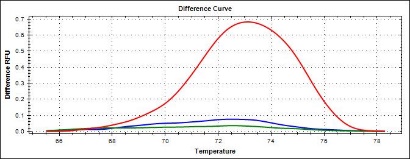


HRM result of sample no.2 represented wild-type *NPM1*


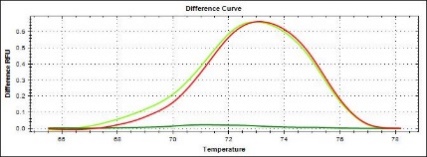


HRM result of sample no.3 represented mutation


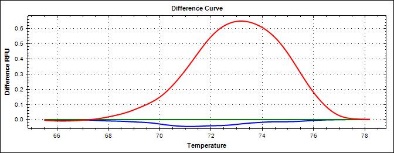


HRM result of sample no.4 represented wild-type *NPM1*


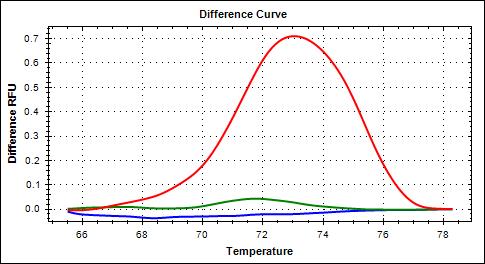


HRM result of sample no.5 represented wild-type *NPM1*


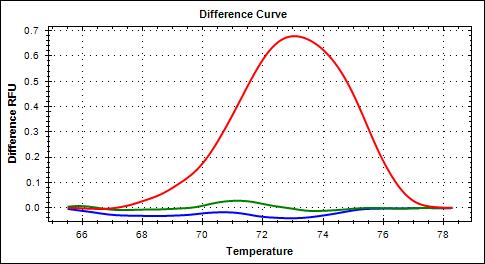


HRM result of sample no.6 represented wild-type *NPM1*


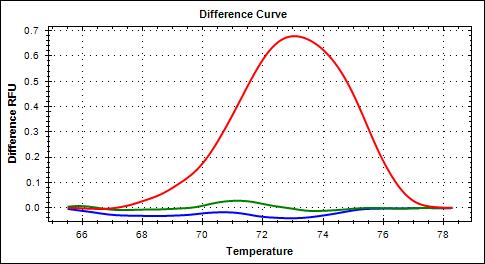


HRM result of sample no.7 represented wild-type *NPM1*


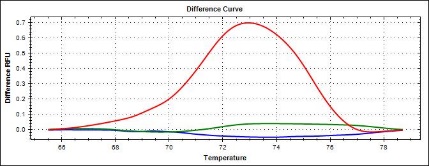


HRM result of sample no.8 represented wild-type *NPM1*


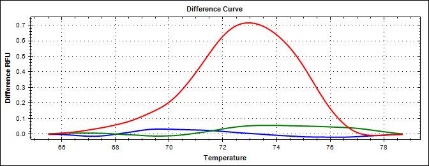


HRM result of sample no.9 represented wild-type *NPM1*


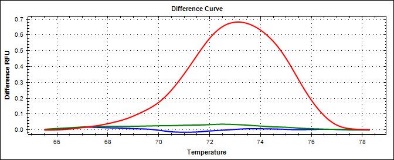


HRM result of sample no.10 represented wild-type *NPM1*


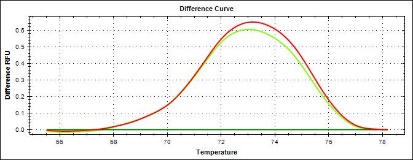


HRM result of sample no.11 represented mutation


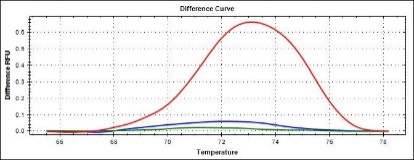


HRM result of sample no.12 represented wild-type *NPM1*


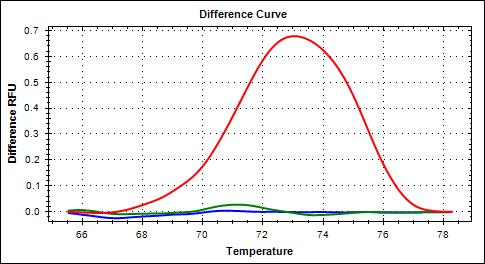


HRM result of sample no.13 represented wild-type *NPM1*


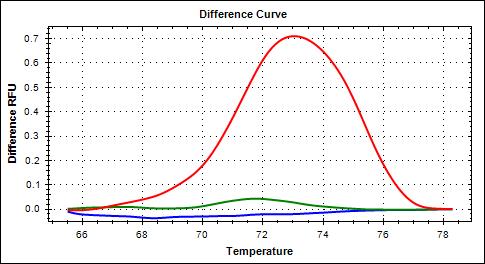


HRM result of sample no.14 represented wild-type *NPM1*


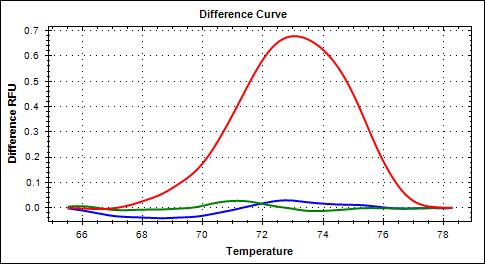


HRM result of sample no.15 represented wild-type *NPM1*


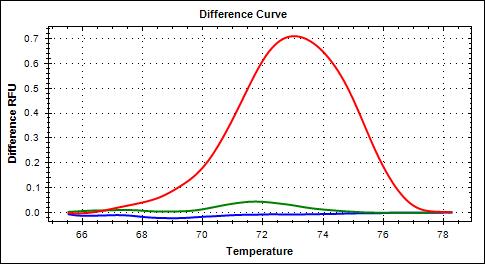


HRM result of sample no.16 represented wild-type *NPM1*


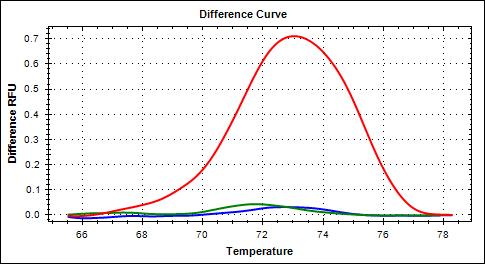


HRM result of sample no.17 represented wild-type *NPM1*


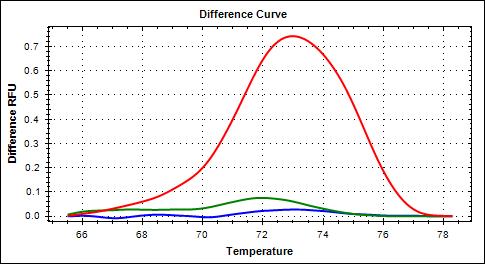


HRM result of sample no.18 represented wild-type *NPM1*


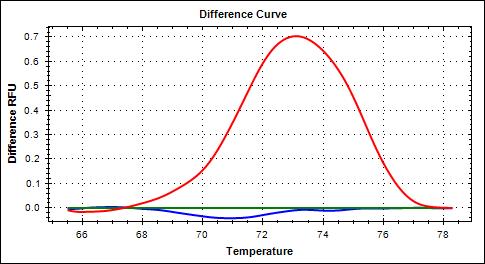


HRM result of sample no.19 represented wild-type *NPM1*


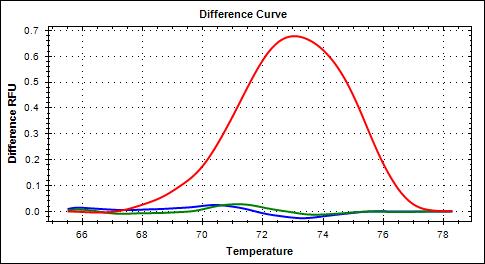


HRM result of sample no.20 represented wild-type *NPM1*


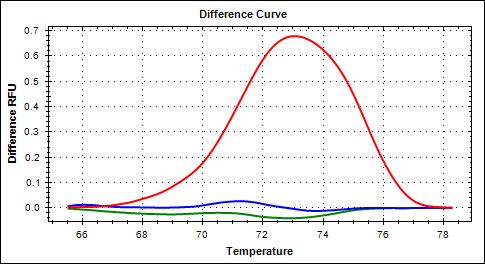


HRM result of sample no.21 represented wild-type *NPM1*


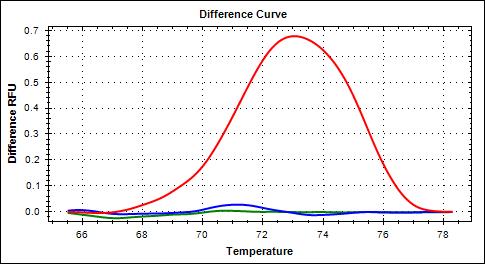


HRM result of sample no.22 represented wild-type *NPM1*


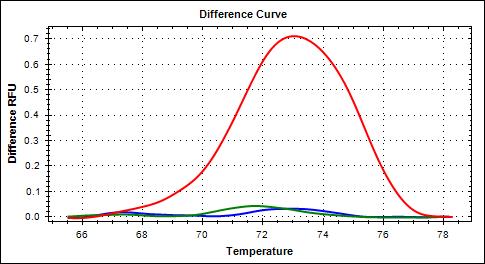


HRM result of sample no.23 represented wild-type *NPM1*


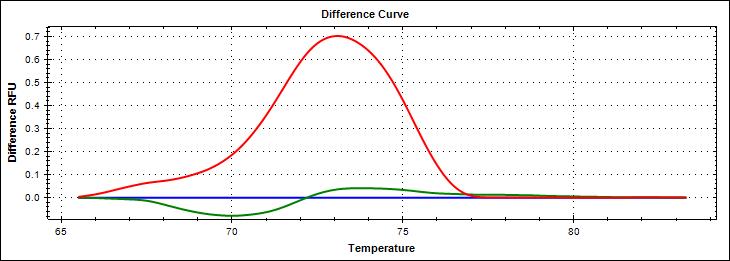


HRM result of sample no.24 represented wild-type *NPM1*


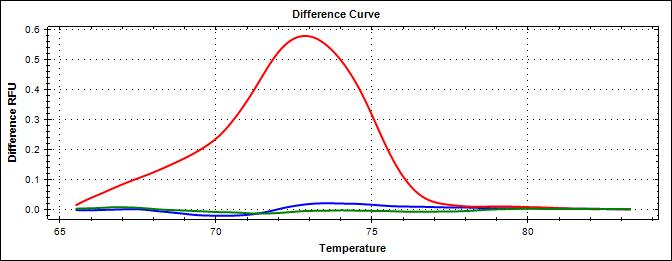


HRM result of sample no.25 represented wild-type *NPM1*


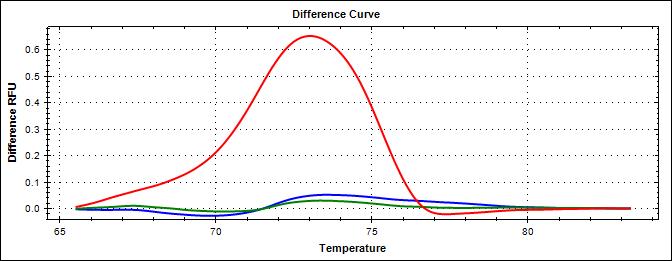


HRM result of sample no.26 represented wild-type *NPM1*


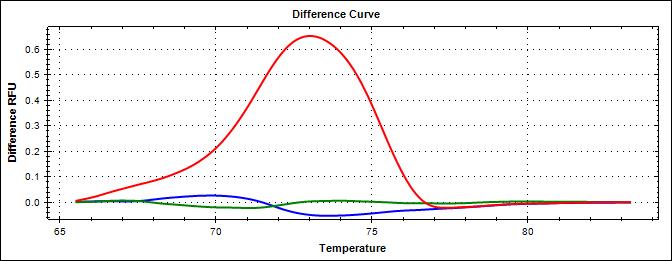


HRM result of sample no.27 represented wild-type *NPM1*


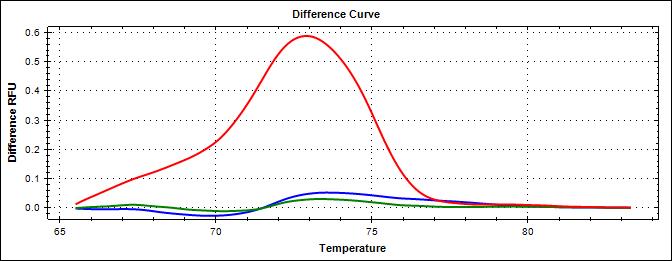


HRM result of sample no.28 represented wild-type *NPM1*


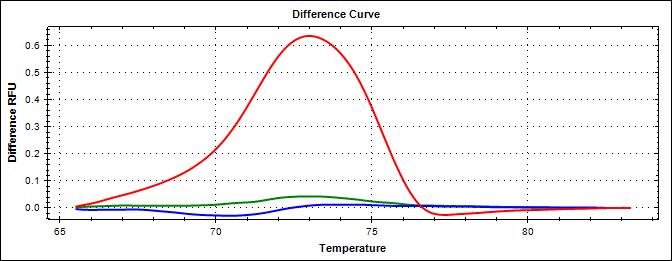


HRM result of sample no.29 represented wild-type *NPM1*


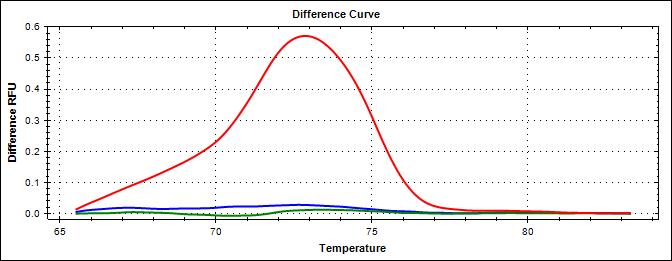


HRM result of sample no.30 represented wild-type *NPM1*


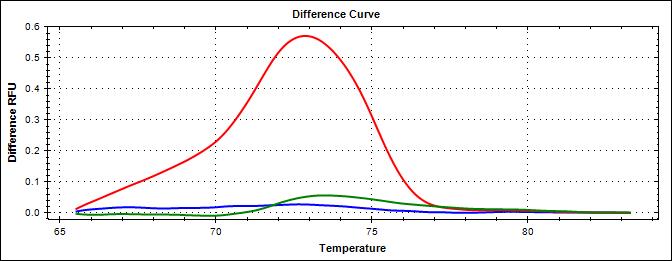


HRM result of sample no.31 represented wild-type *NPM1*


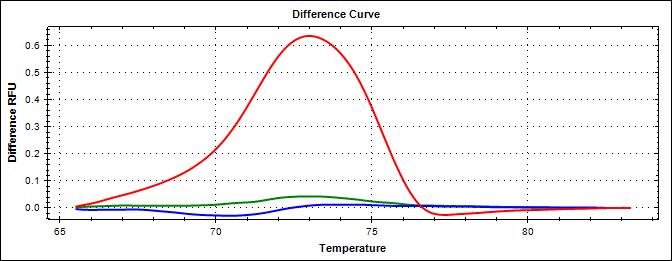


HRM result of sample no.32 represented wild-type *NPM1*


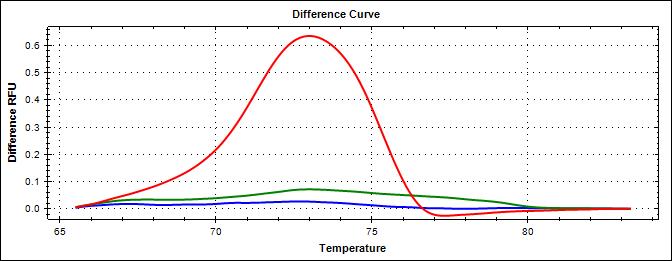


HRM result of sample no.33 represented wild-type *NPM1*


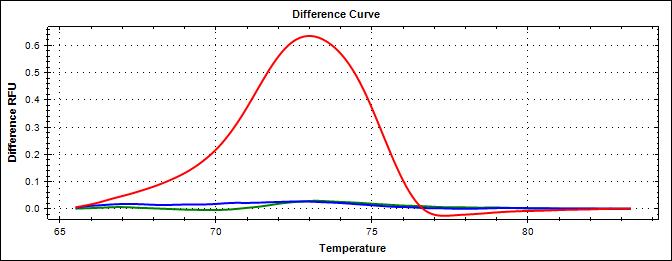


HRM result of sample no.34 represented wild-type *NPM1*


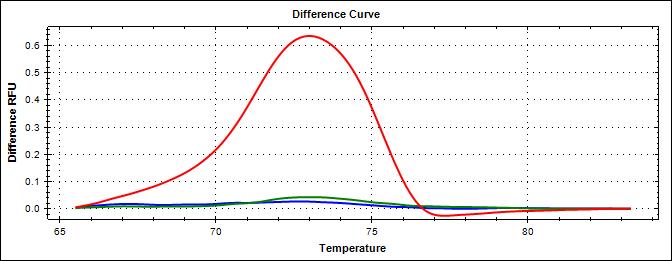


HRM result of sample no.35 represented wild-type *NPM1*


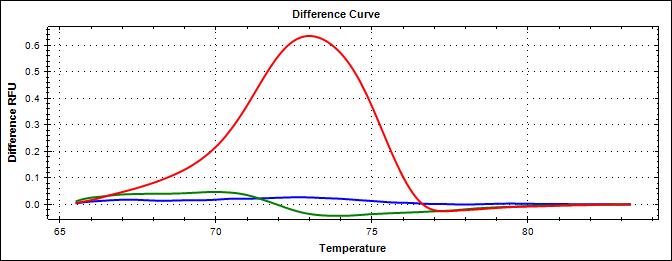


HRM result of sample no.36 represented wild-type *NPM1*


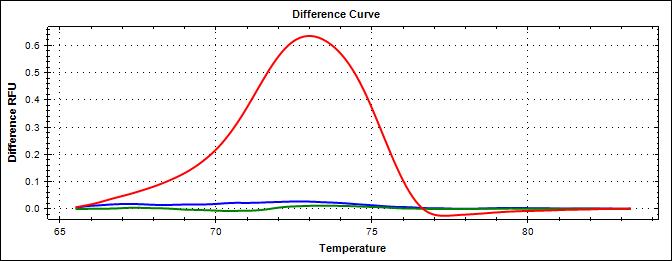


HRM result of sample no.37 represented wild-type *NPM1*


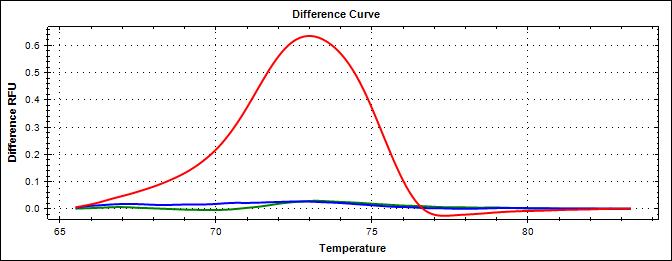


HRM result of sample no.38 represented wild-type *NPM1*


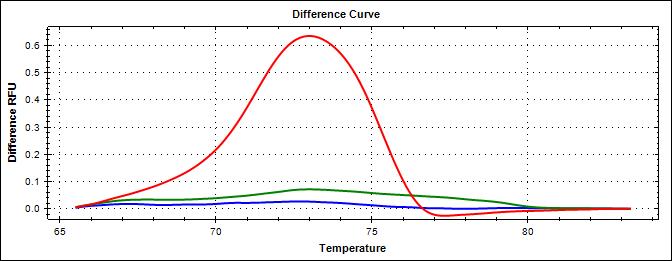


HRM result of sample no.39 represented wild-type *NPM1*


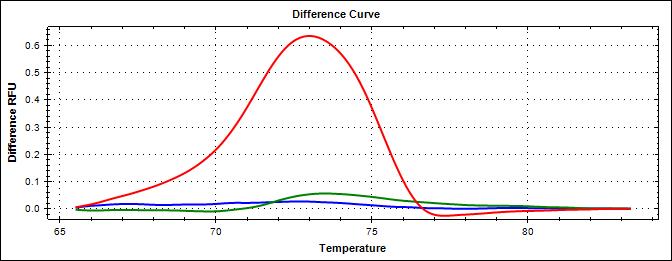


HRM result of sample no.40 represented wild-type *NPM1*


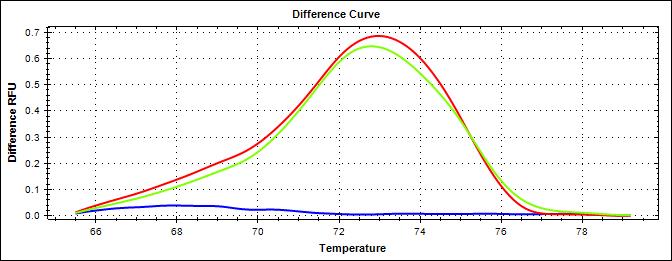


HRM result of sample no.2 represented mutation


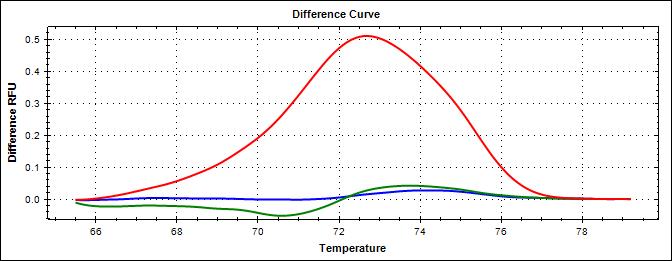


HRM result of sample no.42 represented wild-type *NPM1*


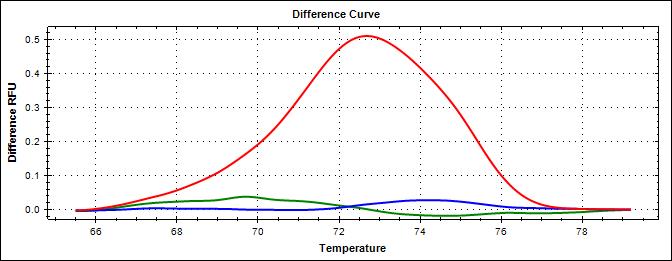


HRM result of sample no.43 represented wild-type *NPM1*


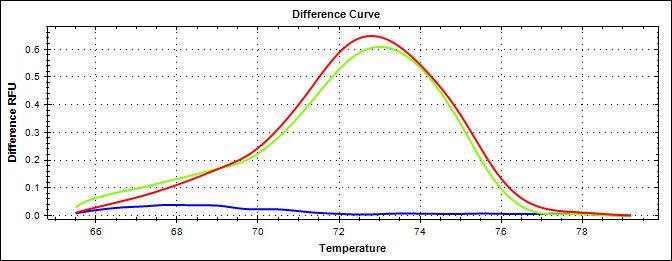


HRM result of sample no.44 represented mutation


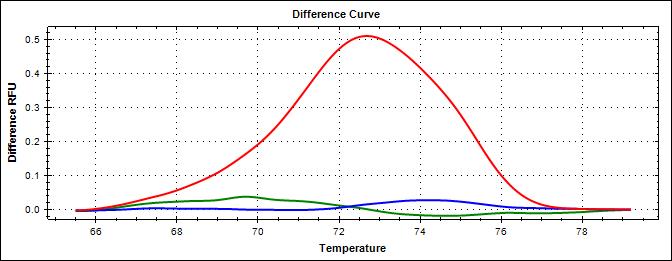


HRM result of sample no.45 represented wild-type *NPM1*


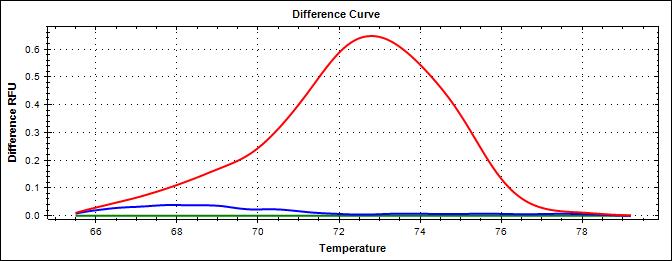


HRM result of sample no.46 represented wild-type *NPM1*


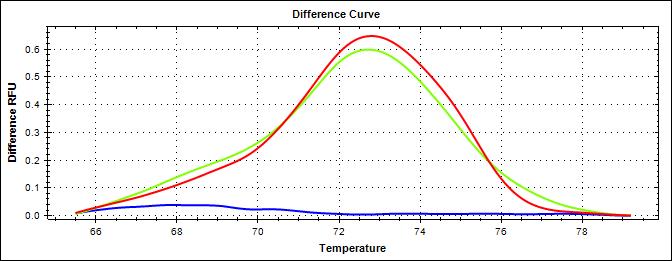


HRM result of sample no.47 represented mutation


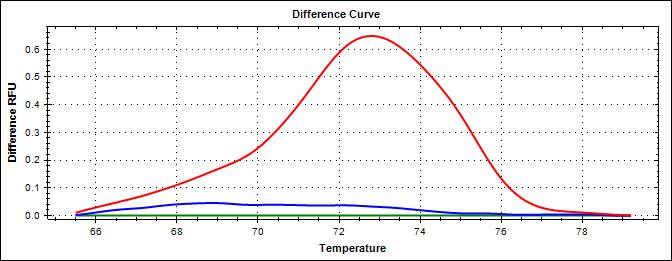


HRM result of sample no.48 represented wild-type *NPM1*


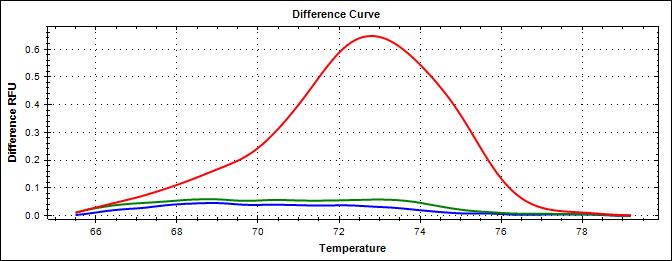


HRM result of sample no.49 represented wild-type *NPM1*


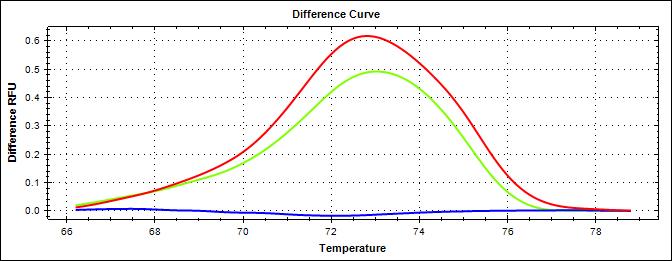


HRM result of sample no.50 represented mutation


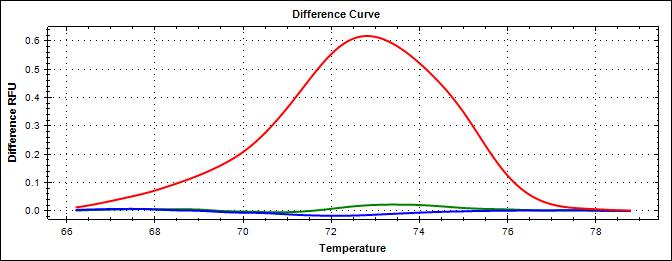


HRM result of sample no.51 represented wild-type *NPM1*


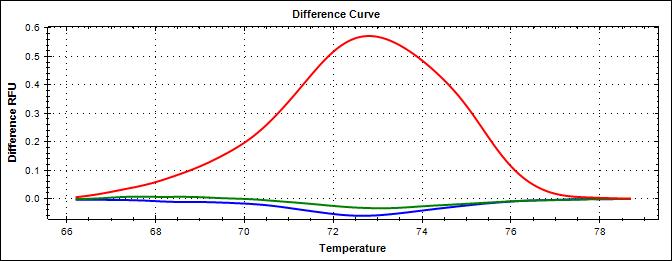


HRM result of sample no.52 represented wild-type *NPM1*


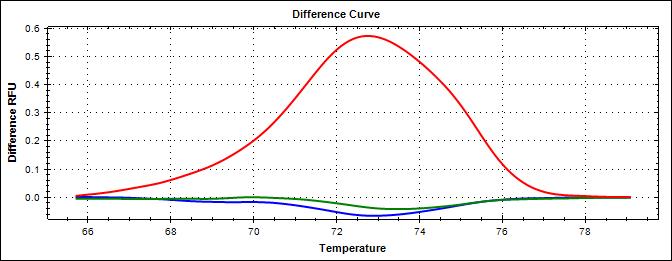


HRM result of sample no.53 represented wild-type *NPM1*


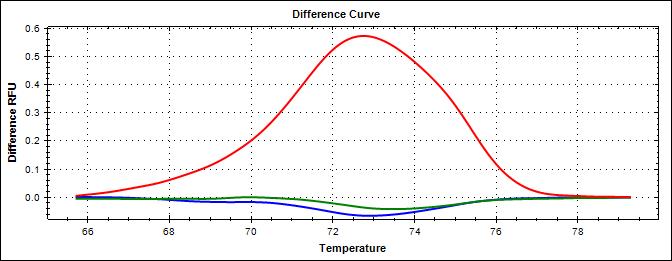


HRM result of sample no.54 represented wild-type *NPM1*


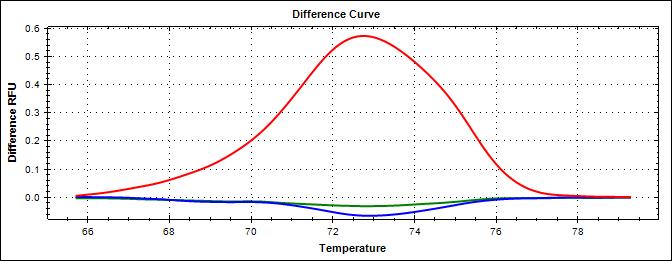


HRM result of sample no.55 represented wild-type *NPM1*


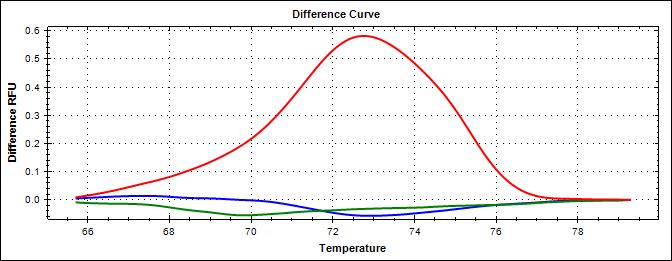


HRM result of sample no.56 represented wild-type *NPM1*


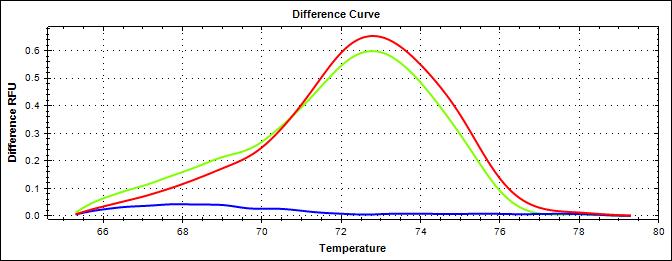


HRM result of sample no.57 represented mutation


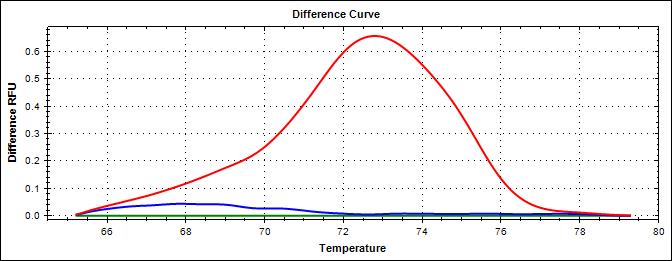


HRM result of sample no.58 represented wild-type *NPM1*


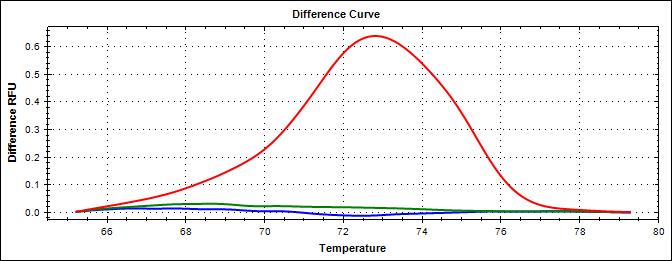


HRM result of sample no.59 represented wild-type *NPM1*


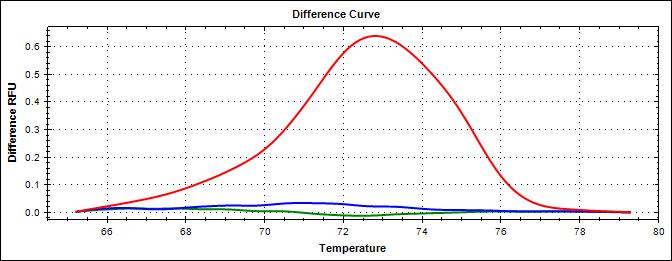


HRM result of sample no.60 represented wild-type *NPM1*


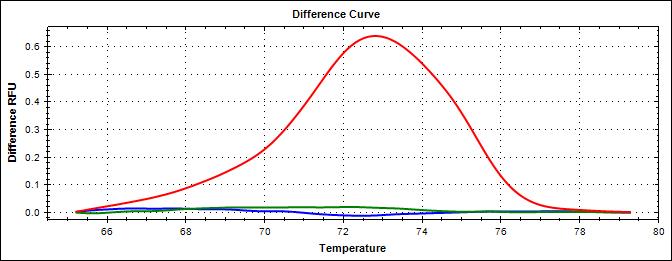


HRM result of sample no.61 represented wild-type *NPM1*


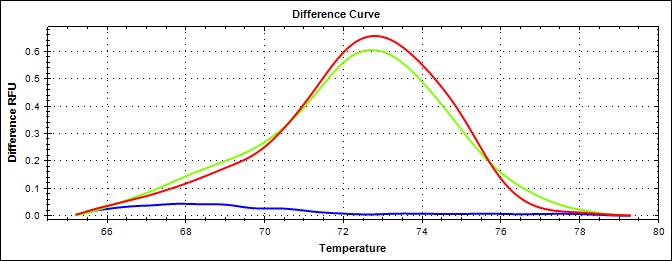


HRM result of sample no.62 represented mutation


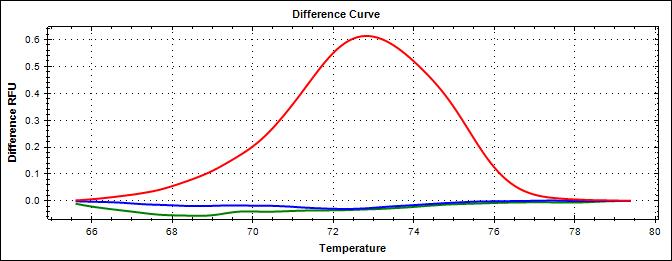


HRM result of sample no.63 represented wild-type *NPM1*


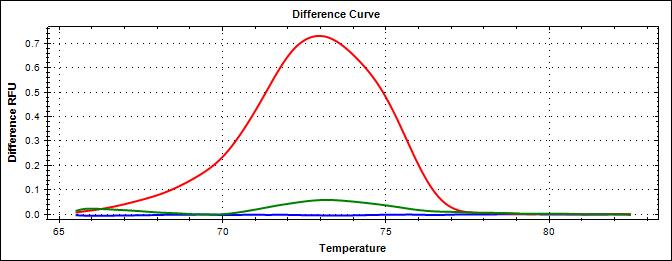


HRM result of sample no.64 represented wild-type *NPM1*


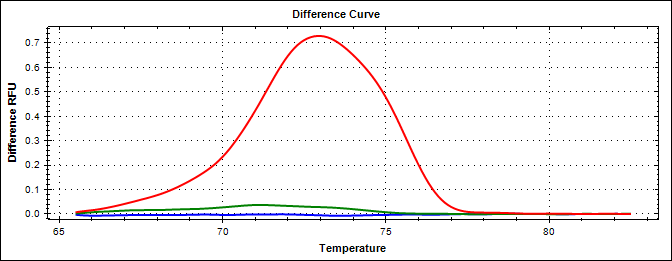


HRM result of sample no.65 represented wild-type *NPM1*


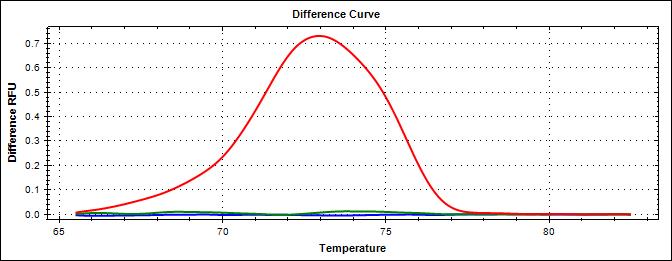


HRM result of sample no.66 represented wild-type *NPM1*


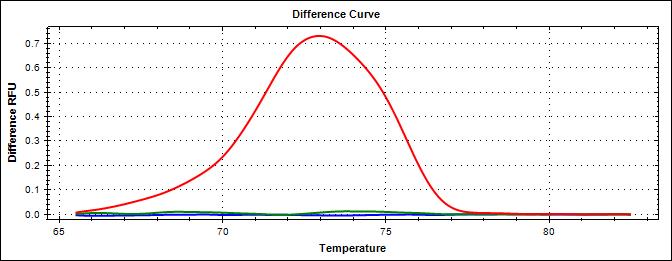


HRM result of sample no.67 represented wild-type *NPM1*


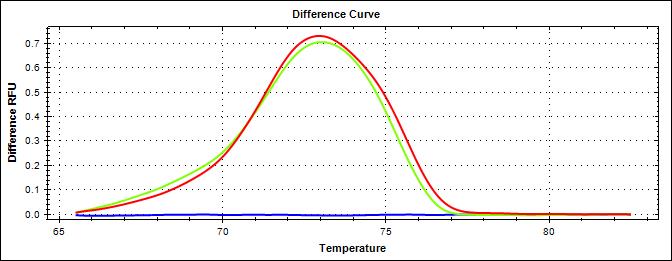


HRM result of sample no.68 represented mutation


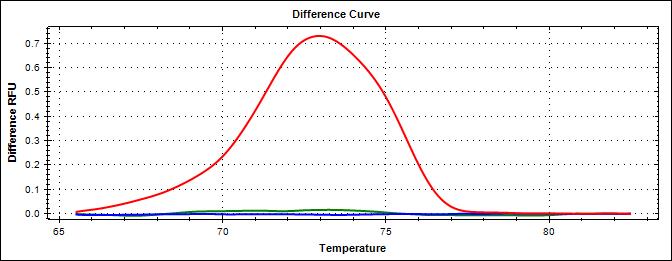


HRM result of sample no.69 represented wild-type *NPM1*


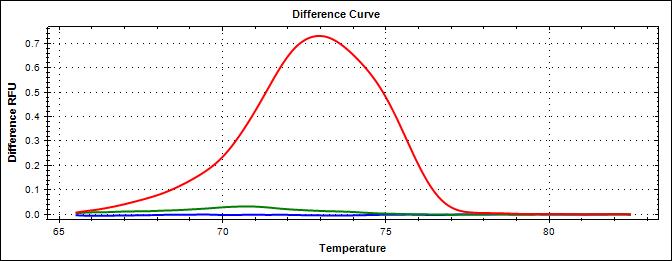


HRM result of sample no.70 represented wild-type *NPM1*


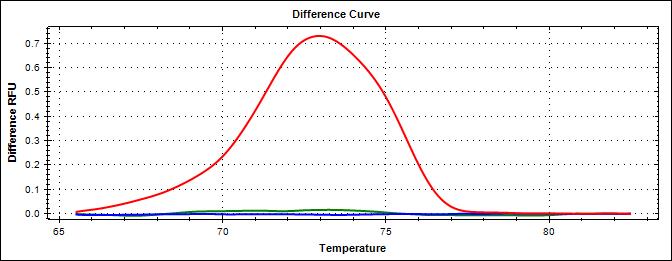


HRM result of sample no.71 represented wild-type *NPM1*


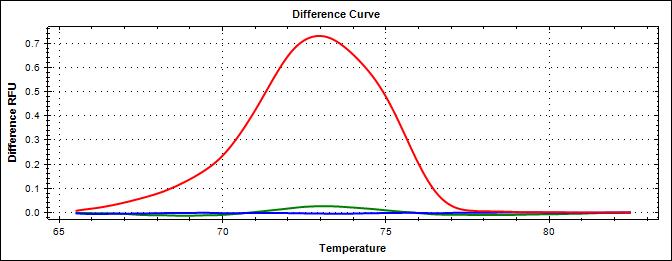


HRM result of sample no.72 represented wild-type *NPM1*


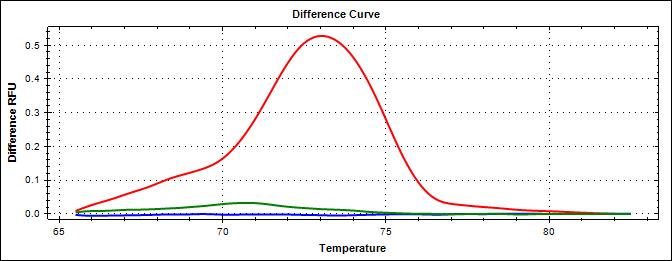


HRM result of sample no.73 represented wild-type *NPM1*


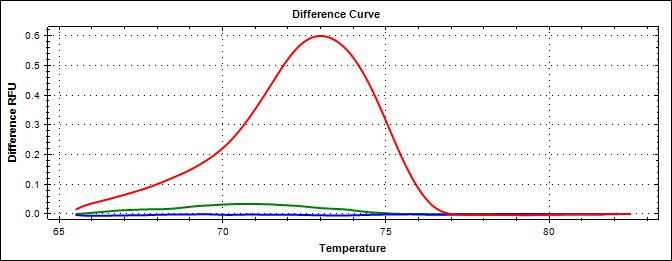


HRM result of sample no.74 represented wild-type *NPM1*


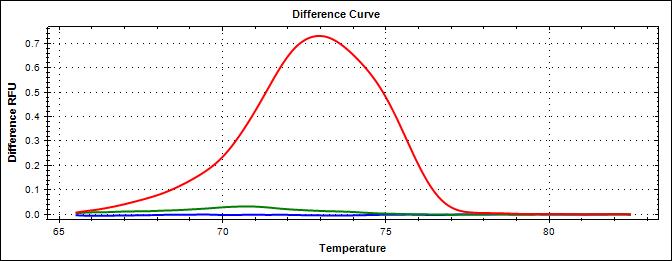


HRM result of sample no.75 represented wild-type *NPM1*


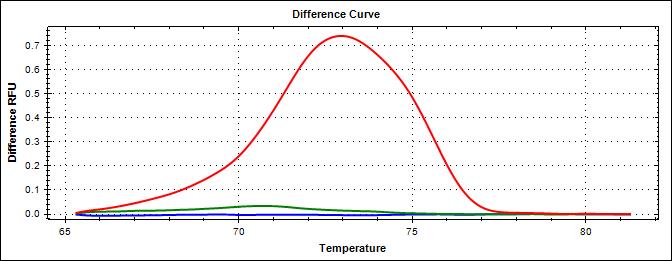


HRM result of sample no.76 represented wild-type *NPM1*


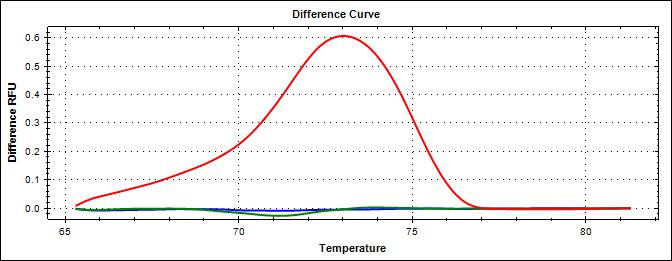


HRM result of sample no.77 represented wild-type *NPM1*


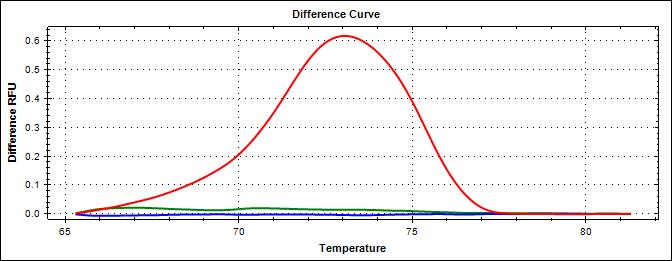


HRM result of sample no.78 represented wild-type *NPM1*


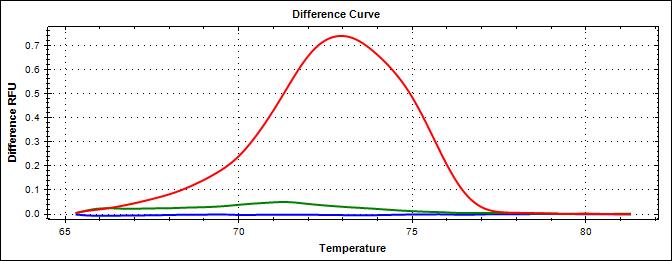


HRM result of sample no.79 represented wild-type *NPM1*


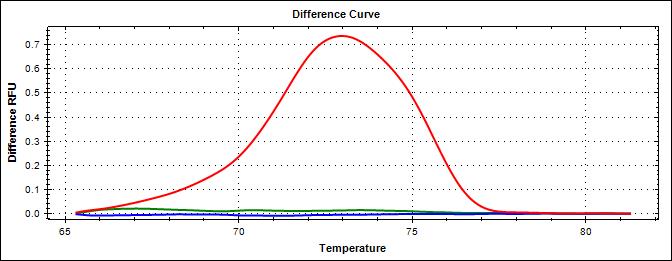


HRM result of sample no.80 represented wild-type *NPM1*


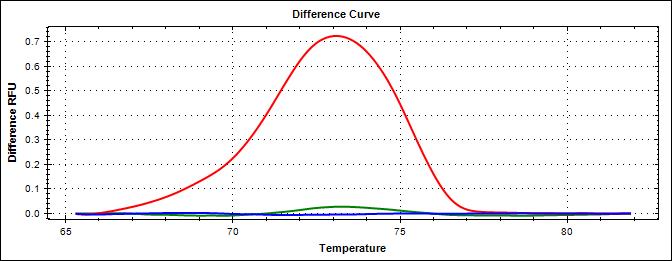


HRM result of sample no.81 represented wild-type *NPM1*


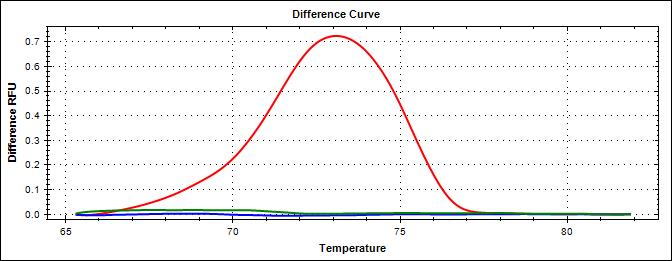


HRM result of sample no.82 represented wild-type *NPM1*


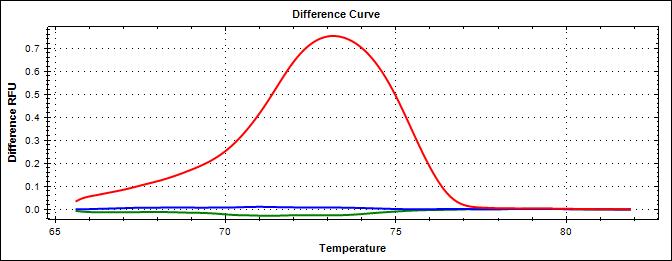


HRM result of sample no.83 represented wild-type *NPM1*
