# Supplementary material for: Development of E-ice-COLD-PCR assay combined with HRM analysis for Nucleophosmin1 gene mutation detection in acute myelogenous leukemia
Source: PLoS One. 2022 Sep 14;17(9):e0274034. doi: 10.1371/journal.pone.0274034 (PMC9473412; doi:10.1371/journal.pone.0274034)
Supplement: S1 Raw images — (PDF) [file pone.0274034.s004.pdf]

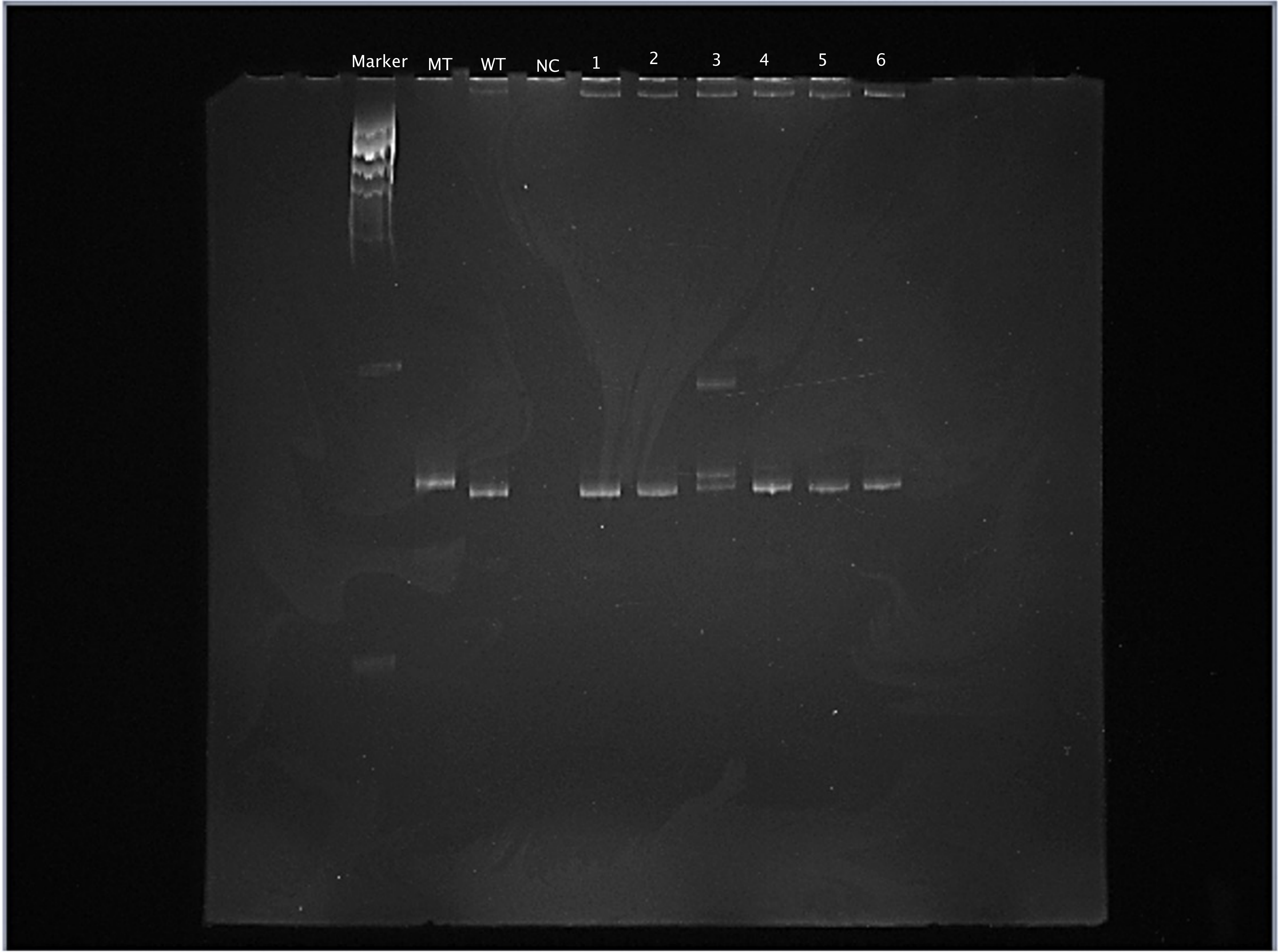
Marker
MT
WT
NC
1
2
3
4
5
6

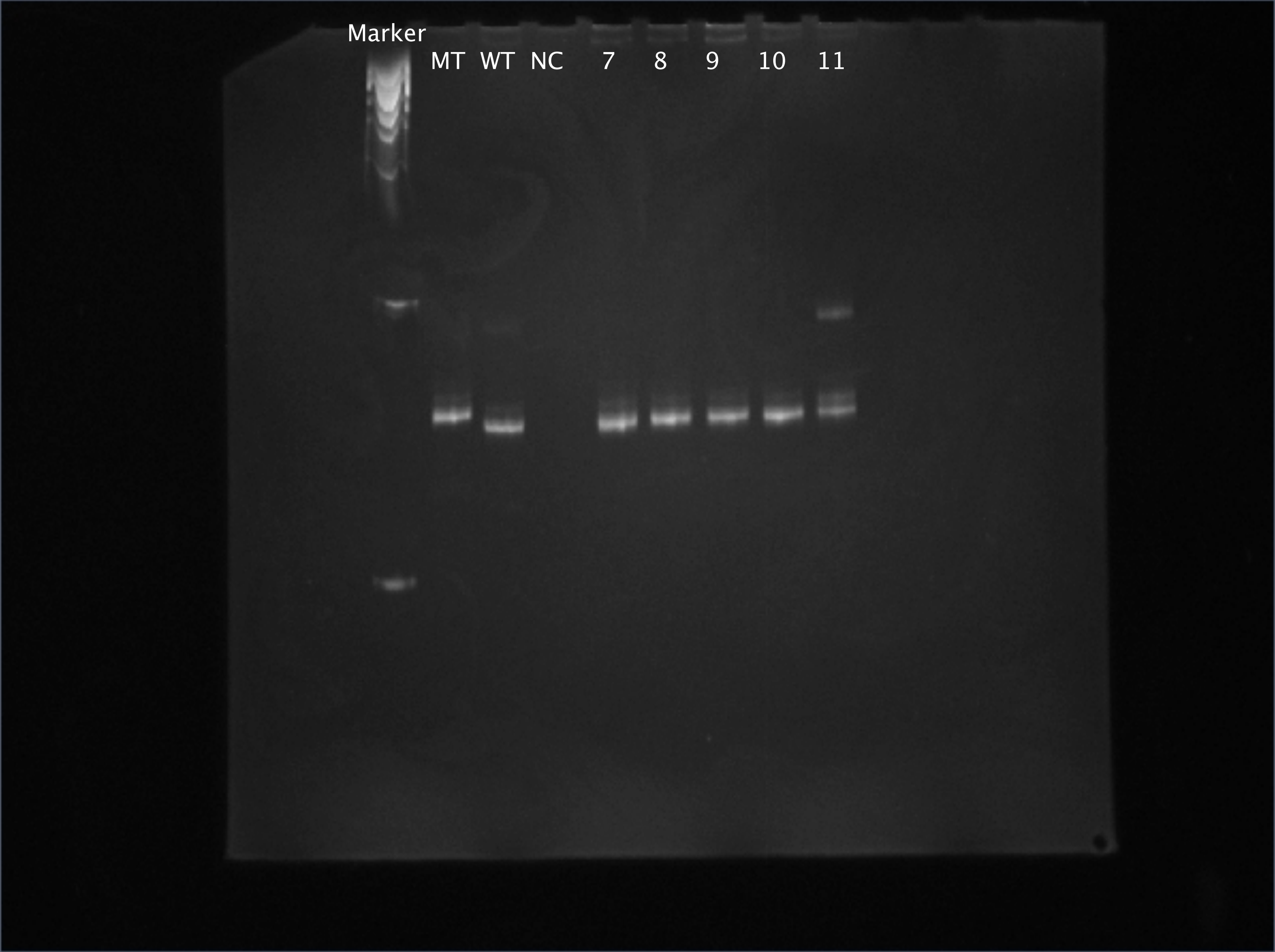
Marker
MT
WT
NC
7
8
9
10
11

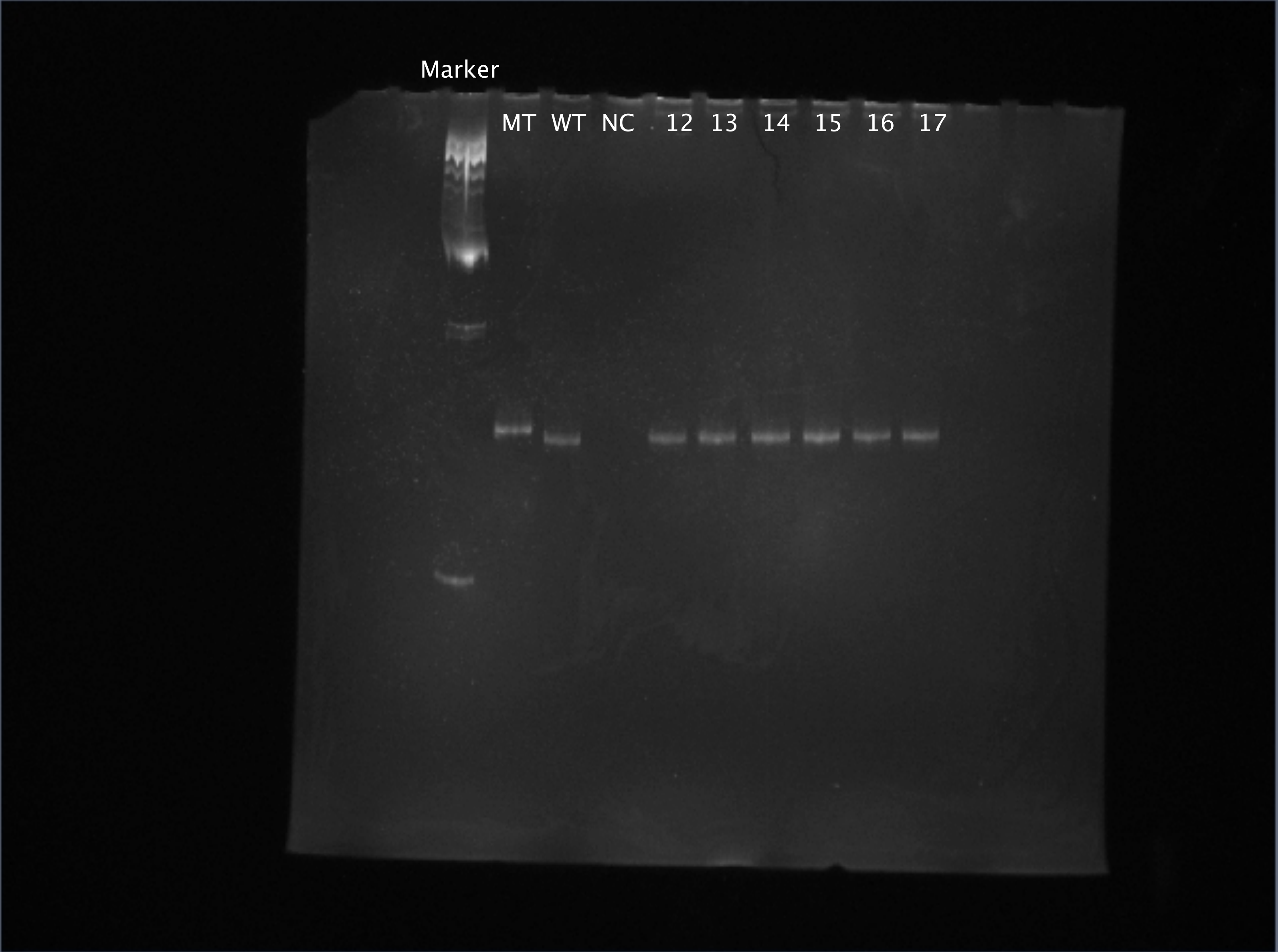
Marker
MT
WT
NC
12
13
14
15
16
17

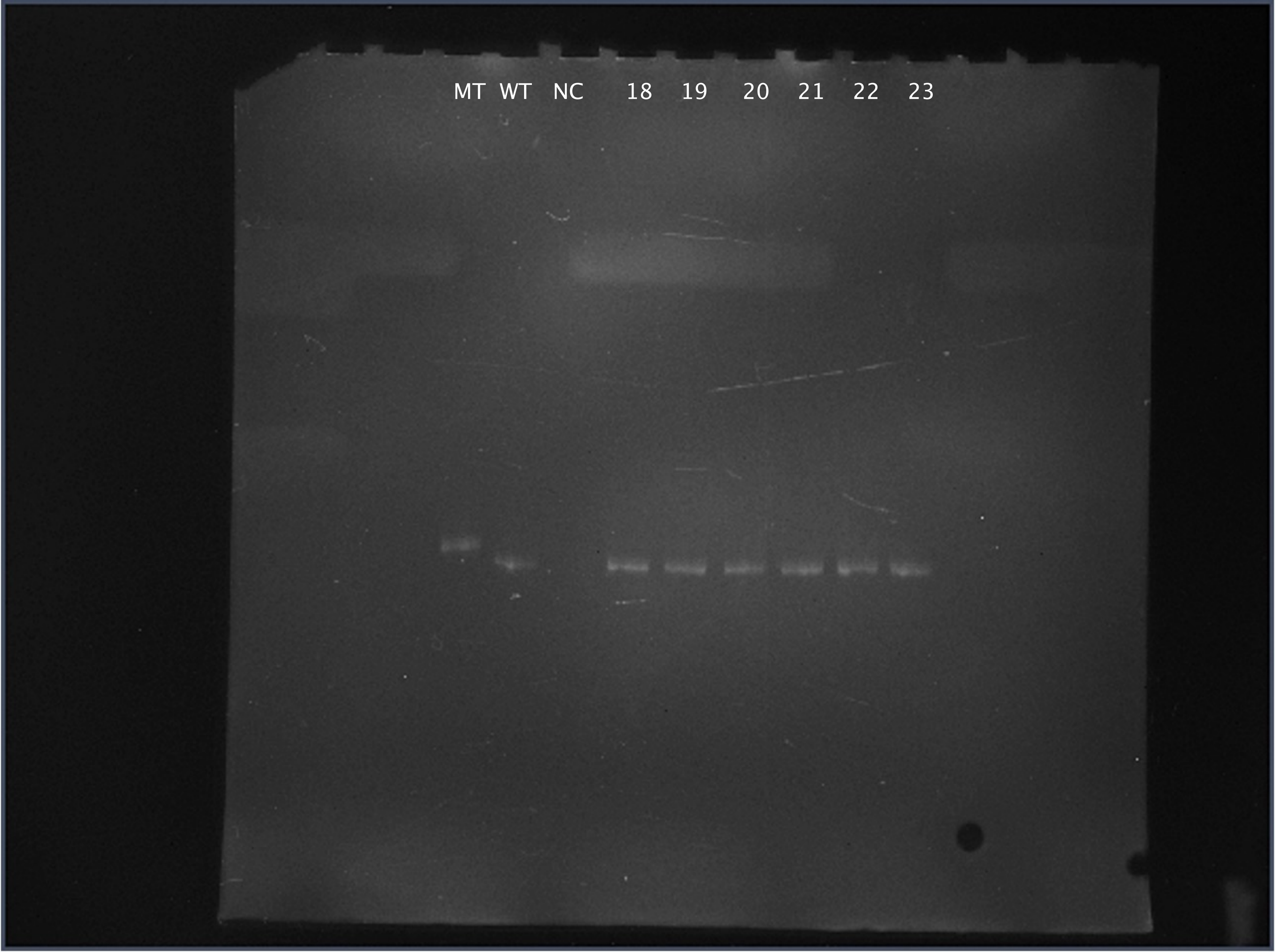
MT WT NC 18 19 20 21 22 23

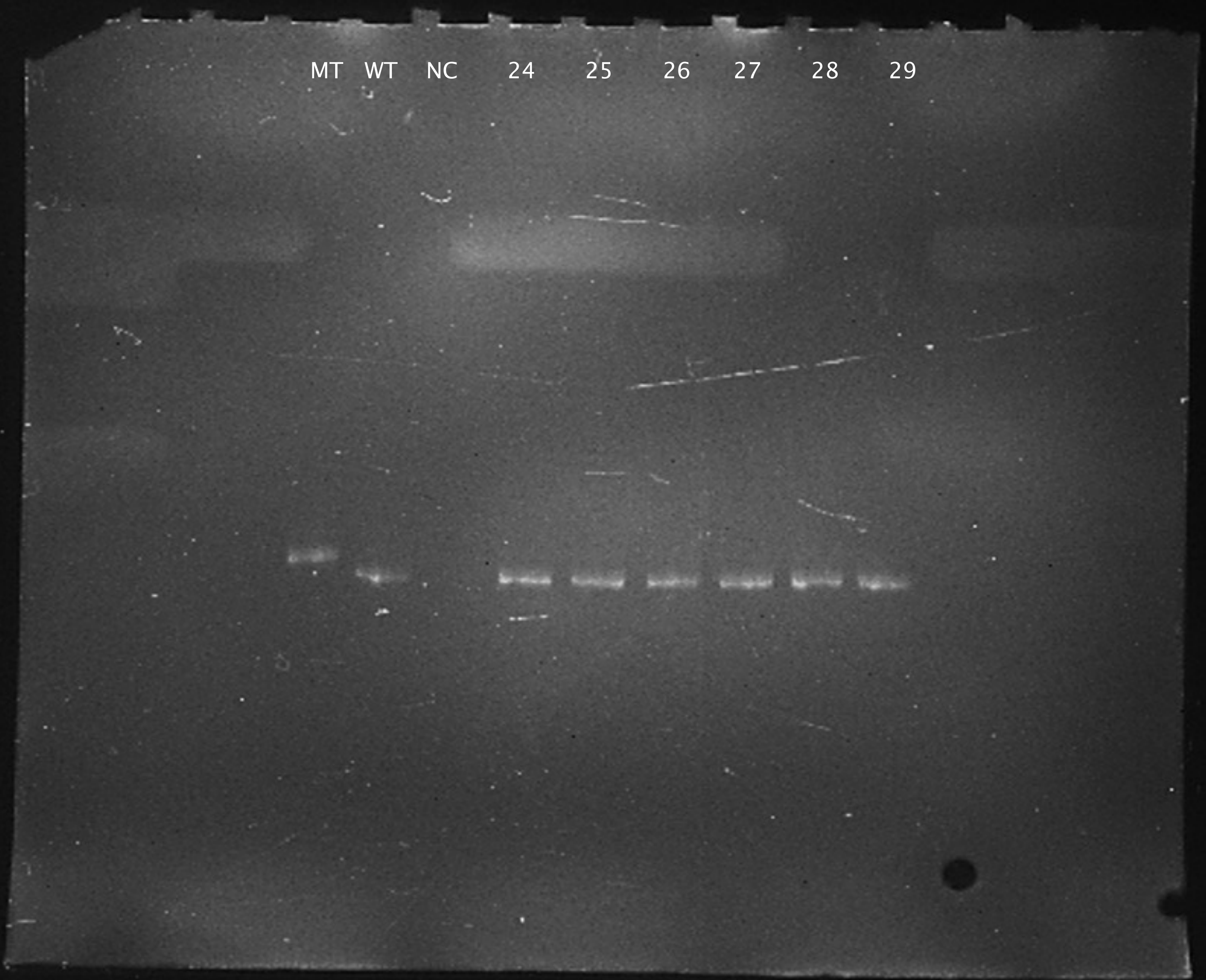
MT WT NC 24 25 26 27 28 29

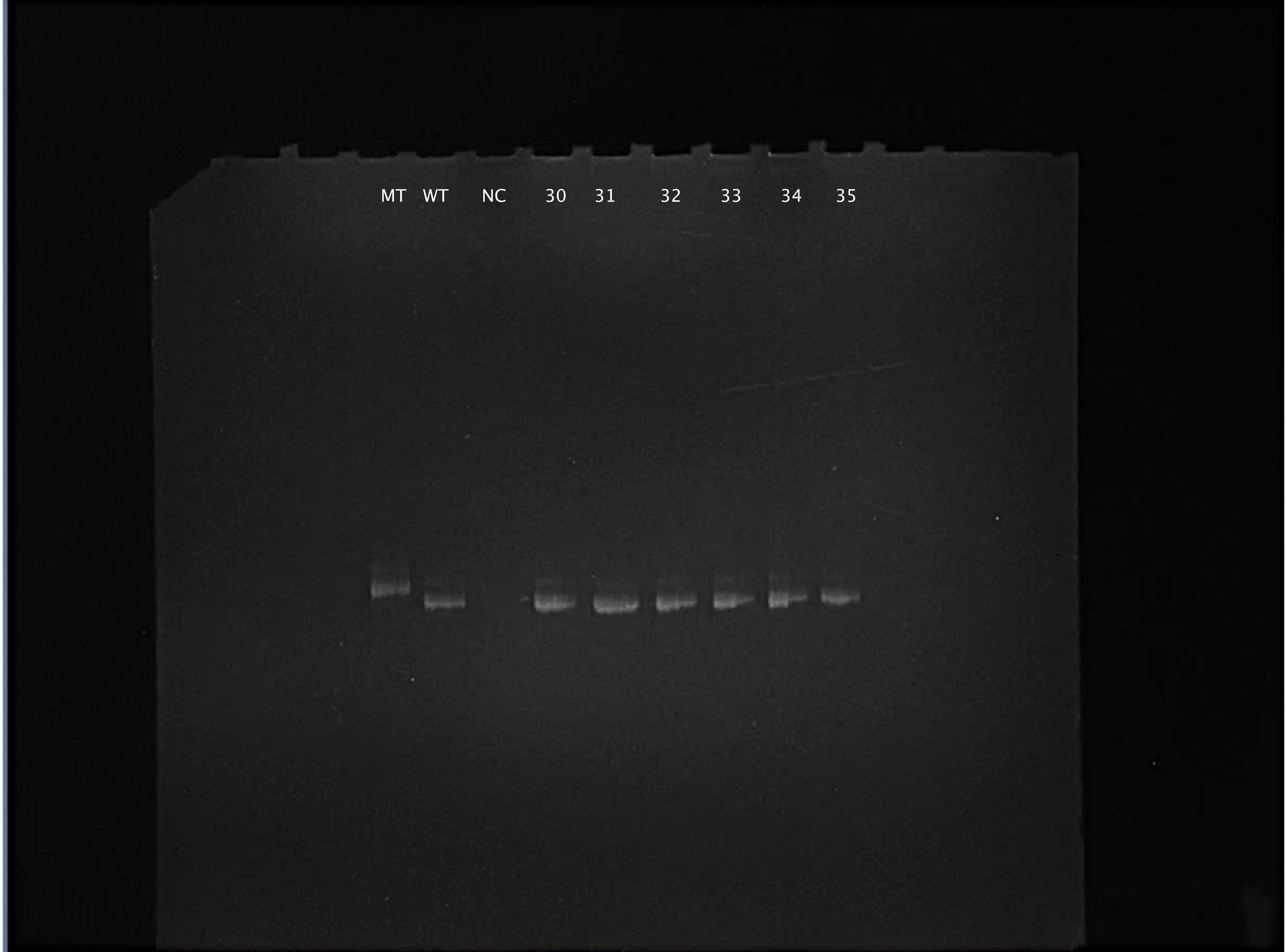
MT
WT
NC
30
31
32
33
34
35

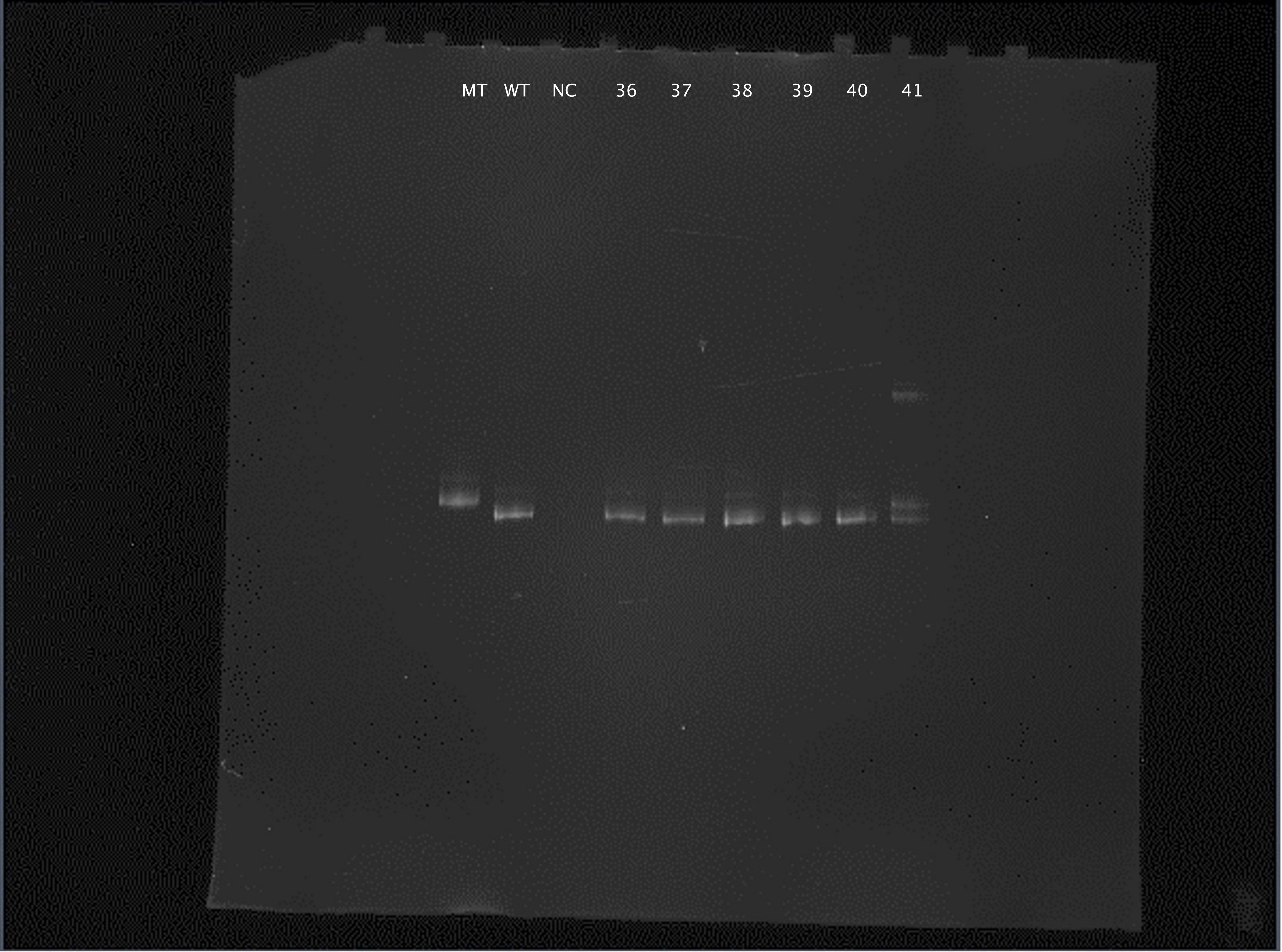
MT
WT
NC
36
37
38
39
40
41

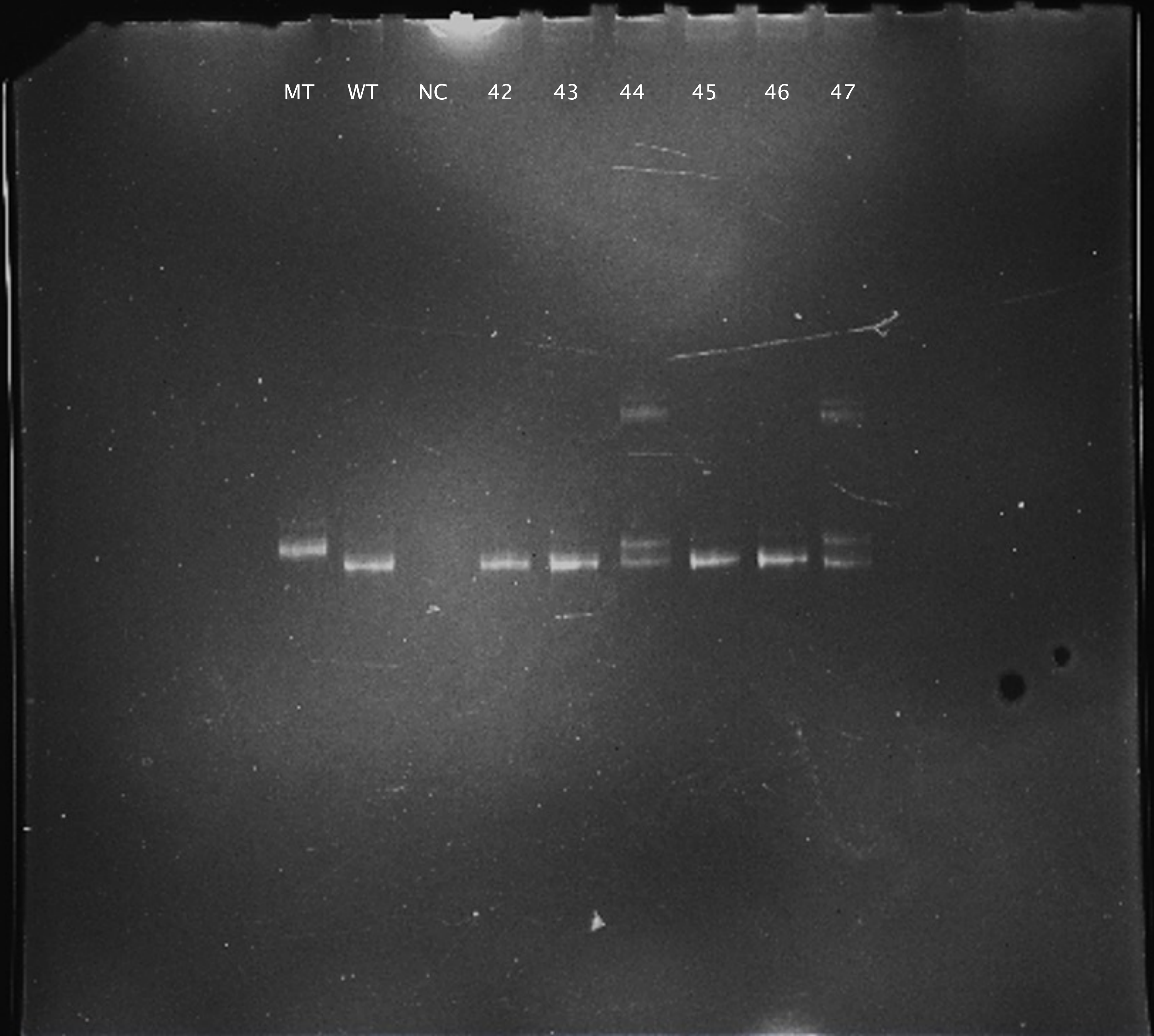
MT
WT
NC
42
43
44
45
46
47

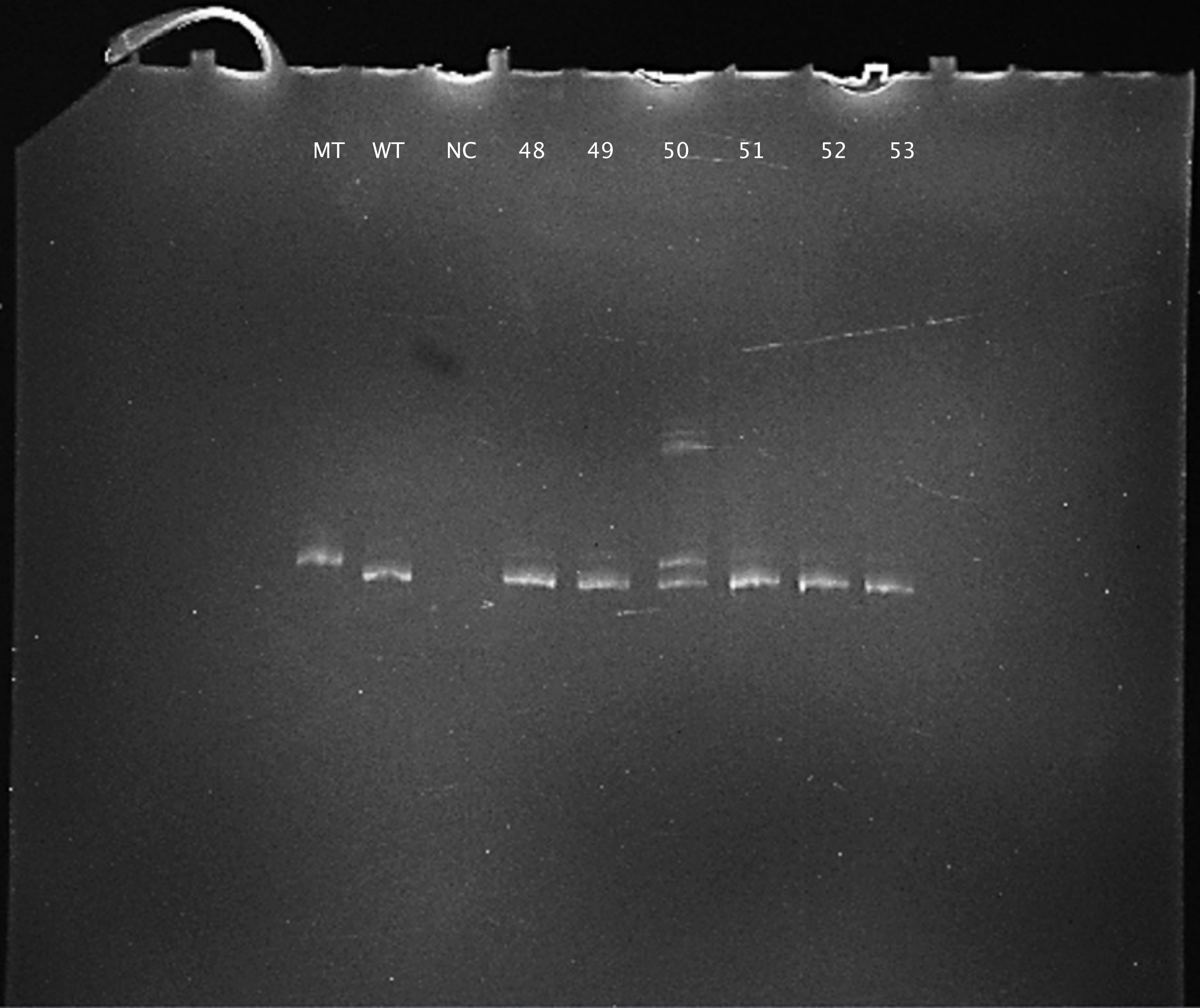
MT WT NC 48 49 50 51 52 53

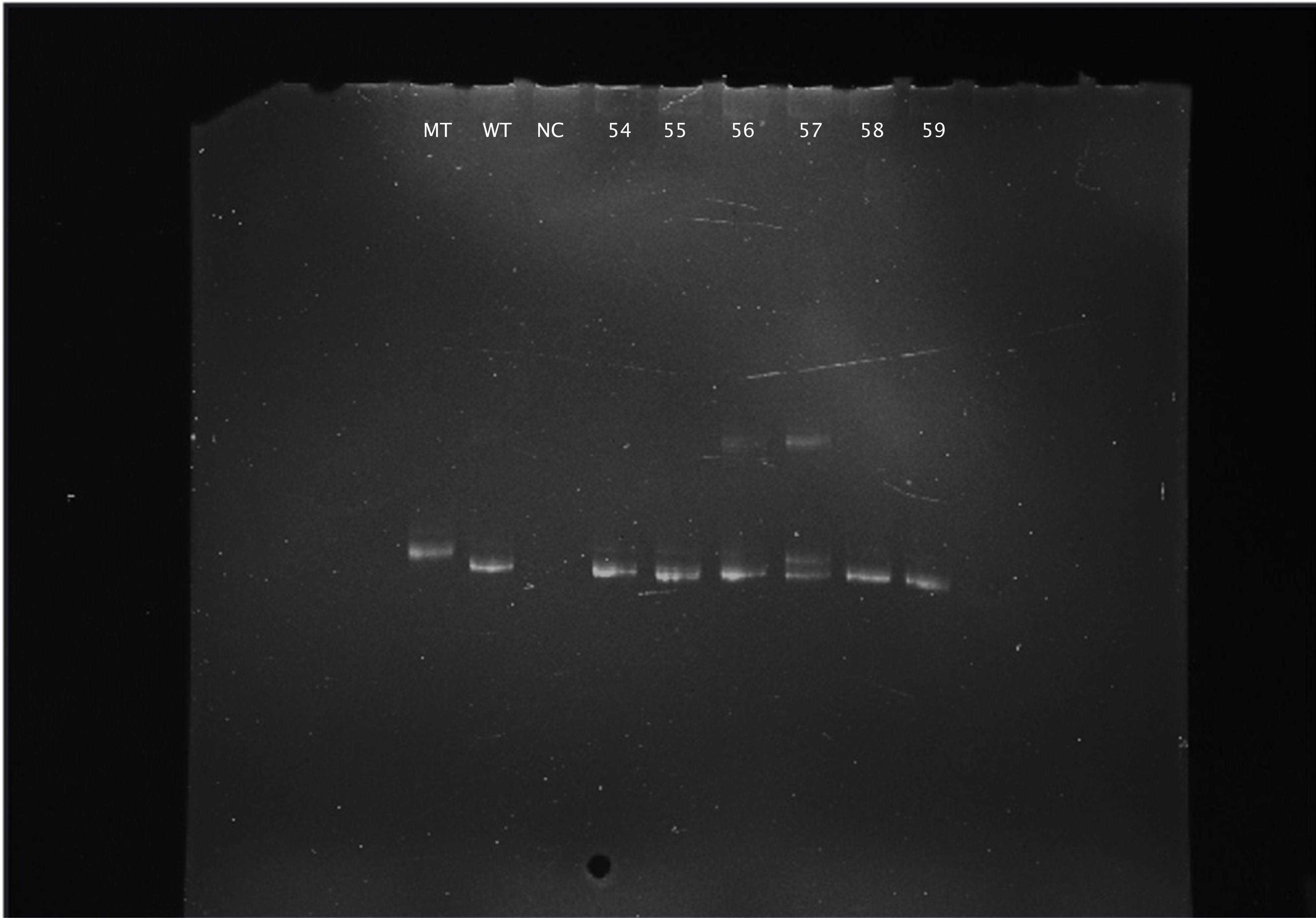
MT
WT
NC
54
55
56
57
58
59

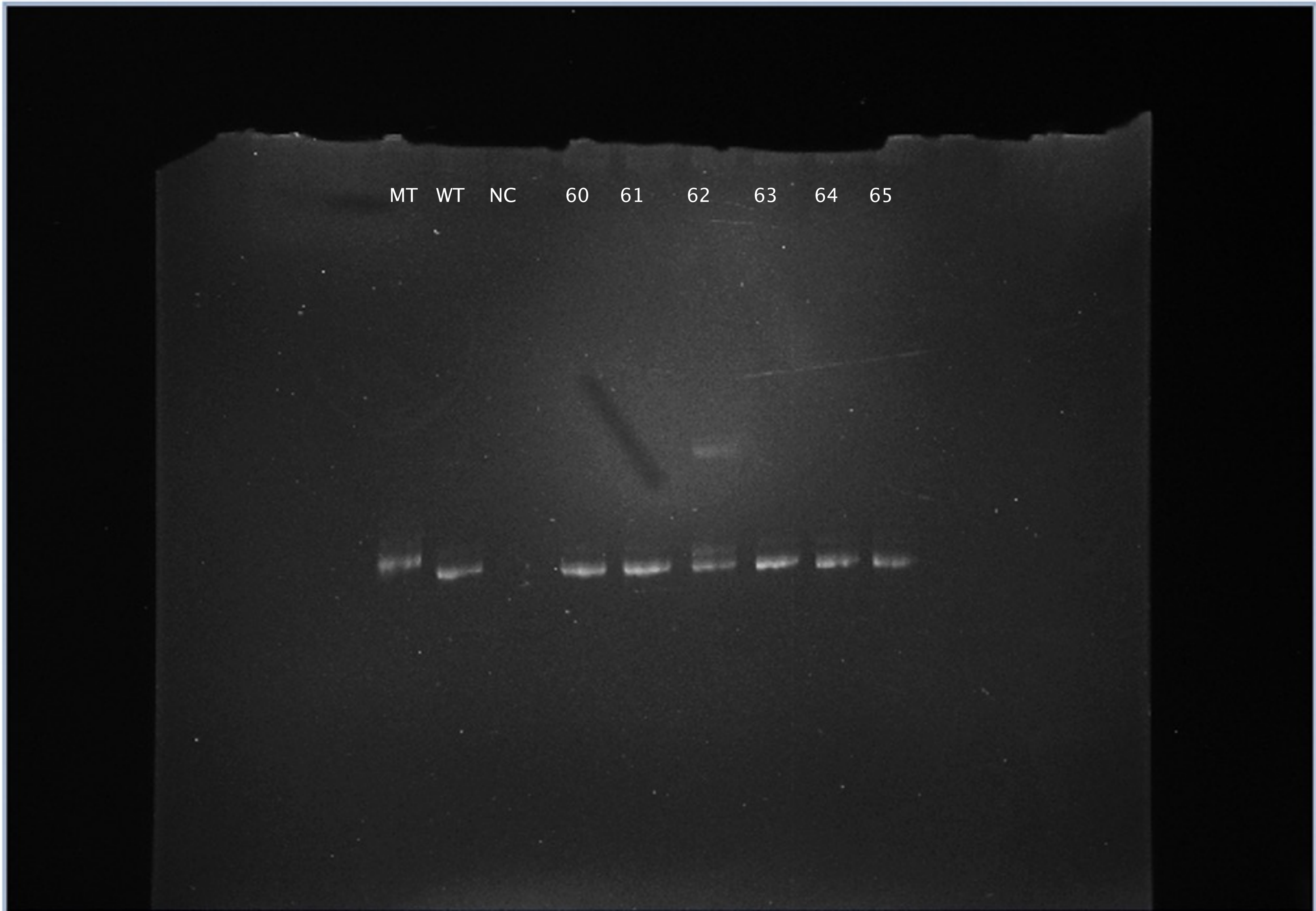
MT
WT
NC
60
61
62
63
64
65

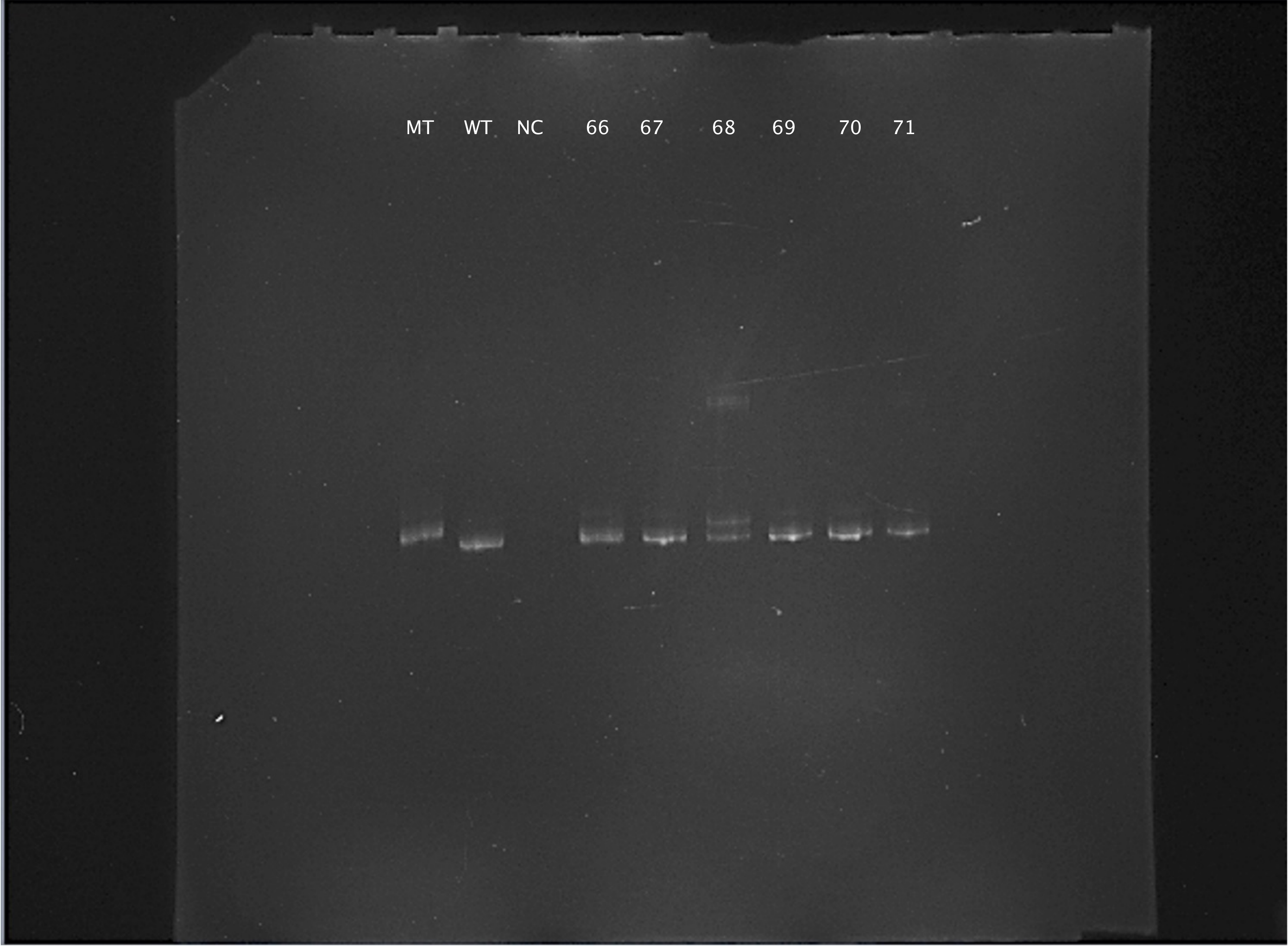
MT
WT
NC
66
67
68
69
70
71

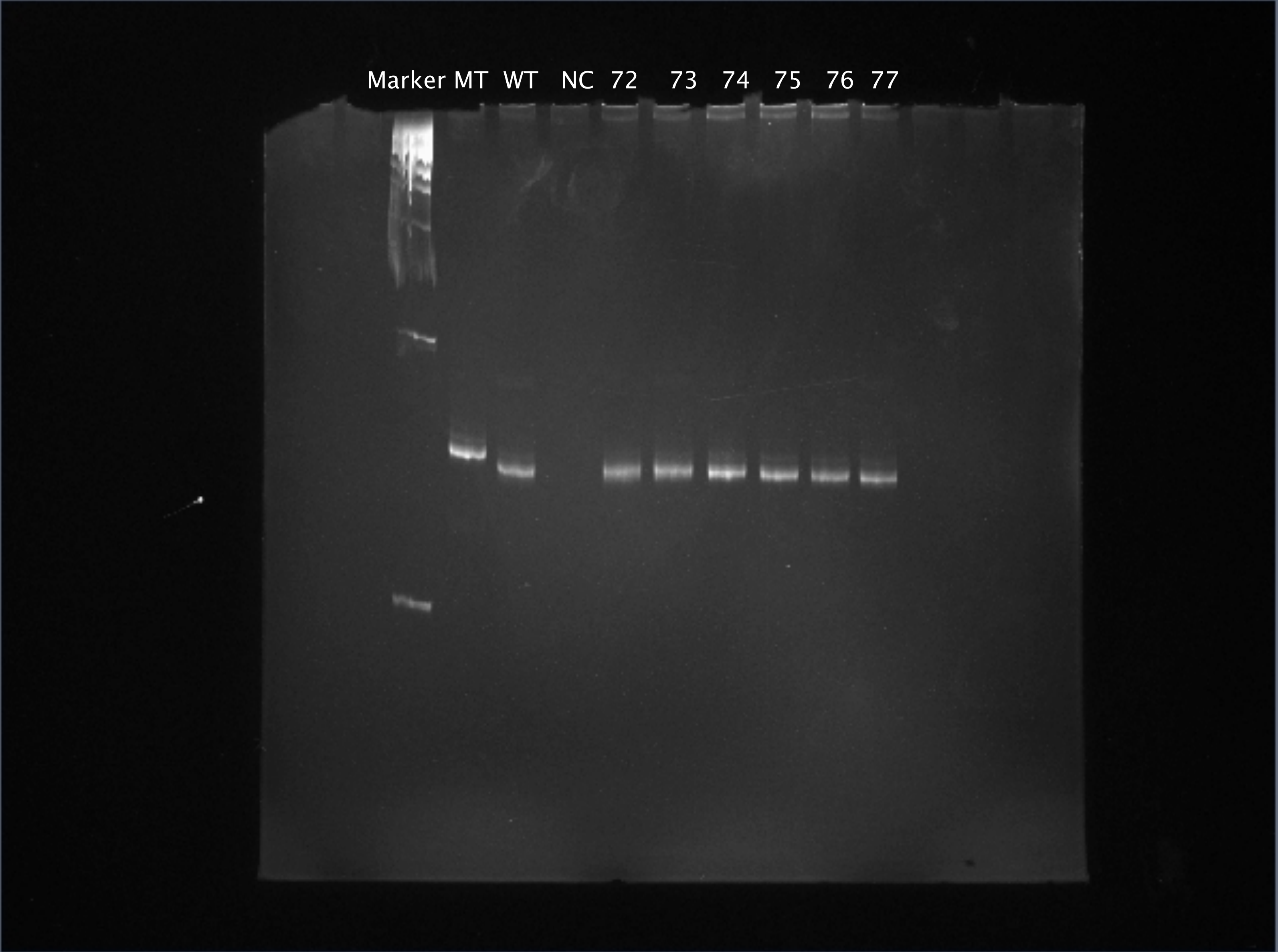
Marker MT WT NC 72 73 74 75 76 77

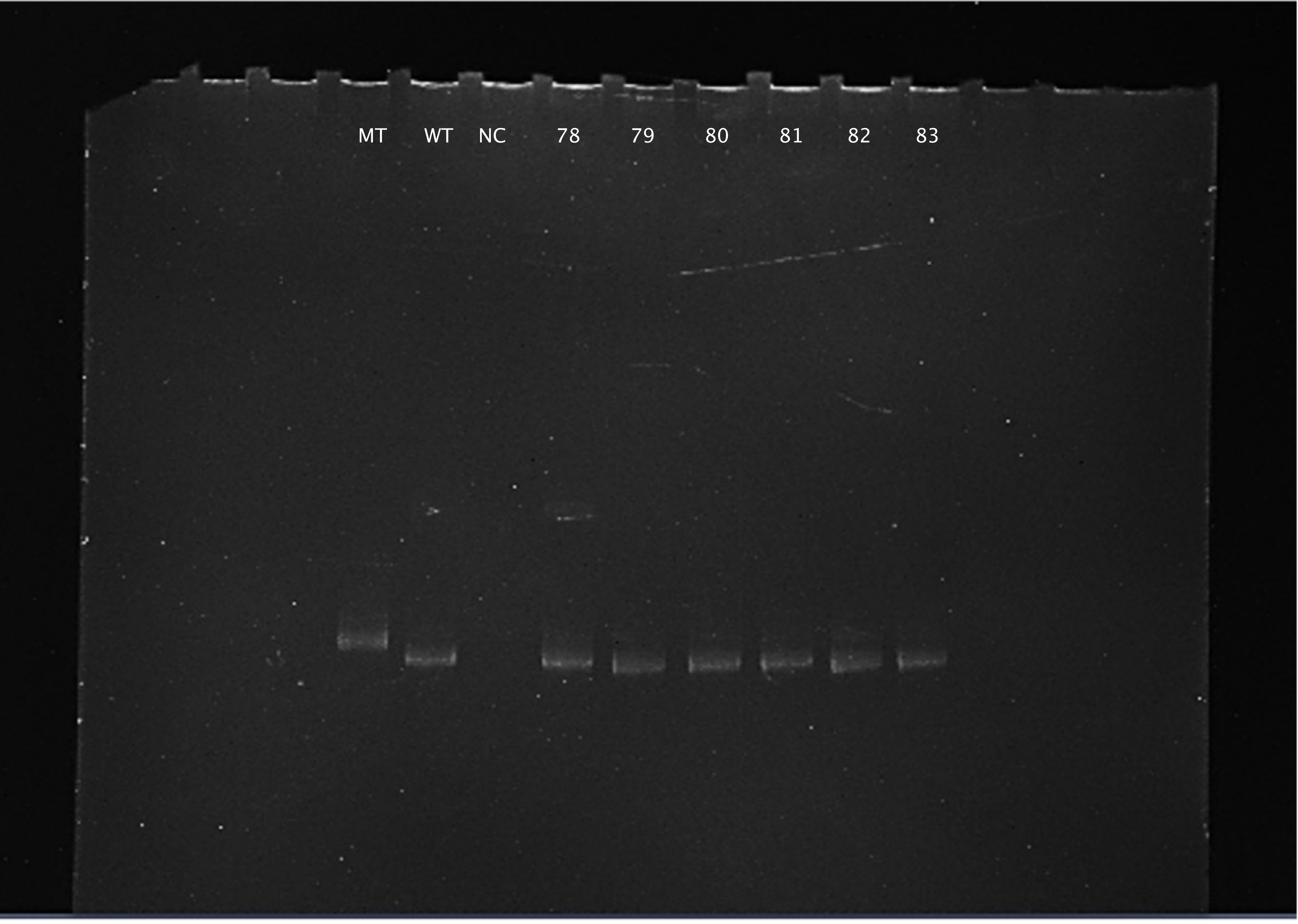
MT
WT
NC
78
79
80
81
82
83
